# Visual and Neuro-Ophthalmic Manifestations of John Cunningham (JC) Virus-Related Natalizumab-Associated Progressive Multifocal Leukoencephalopathy in Multiple Sclerosis: A Systematic Review

**DOI:** 10.7759/cureus.110720

**Published:** 2026-06-12

**Authors:** Tornike Jangirashvili, Orchid Parthiv, Lolita Shengelia, Zurab Tabeshadze, Ana Kontchoshvili, Christopher A Waked, Ribal M Tamer

**Affiliations:** 1 Neurology, Georgian American University, Tbilisi, GEO; 2 Neurology, Georgian Youth Health Association, Tbilisi, GEO; 3 Research, Georgian American University, Tbilisi, GEO; 4 Radiology, Georgian American University, Tbilisi, GEO

**Keywords:** jc polyomavirus, multiple sclerosis, natalizumab, neuro-ophthalmology, neuroradiology, progressive multifocal leukoencephalopathy

## Abstract

Natalizumab (Tysabri®; Biogen, Cambridge, Massachusetts), a recombinant humanized monoclonal antibody targeting the α4-integrin subunit, is among the most efficacious approved therapies for relapsing-remitting multiple sclerosis (RRMS). Its principal serious adverse effect is progressive multifocal leukoencephalopathy (PML), an opportunistic demyelinating encephalitis caused by reactivation of the John Cunningham (JC) polyomavirus (JCPyV). Despite the established clinical significance of this complication, its visual and neuro-ophthalmic dimensions have not been systematically synthesized.

To provide a comprehensive, Preferred Reporting Items for Systematic Reviews and Meta-Analyses (PRISMA)-compliant systematic review of the spectrum, prevalence, anatomical substrates, and clinical significance of visual and neuro-ophthalmic manifestations in natalizumab-associated PML, and to synthesize available evidence on risk stratification, magnetic resonance imaging (MRI) correlates, immune reconstitution inflammatory syndrome (IRIS), and long-term functional outcomes.

A systematic literature search was conducted across PubMed/MEDLINE, ScienceDirect, Google Scholar, and ResearchGate (January 2012 to April 2025) in accordance with PRISMA 2020 guidelines. Medical Subject Headings (MeSH) and structured free-text keyword strategies were applied. Eligibility criteria, data extraction, and quality appraisal were predefined. Thirty-five studies were included: 20 observational cohorts or case series, seven systematic reviews or meta-analyses, and eight narrative reviews with extractable data. To prevent double-counting, quantitative outcome data were extracted exclusively from primary observational studies; reviews contributed contextual synthesis only.

Neuro-ophthalmic involvement was documented in 20-50% of natalizumab-associated PML patients across the included studies. Homonymous hemianopia was the most prevalent overt manifestation, arising from lytic demyelination of the optic radiations; occipital lobe involvement was recorded in 20% of cases in the largest dedicated MRI distribution dataset. Visual symptoms constituted the initial presentation in up to 25% of affected individuals. Subclinical visual field deficits were identified in 17.4% of post-PML survivors by formal perimetry in the absence of spontaneous visual complaint. Asymptomatic MRI-detected PML was associated with a modified Rankin scale score of two or below at follow-up in 64% of patients, compared with 34% among those diagnosed after symptom onset (p = 0.012). IRIS developed in 57-69% of patients following natalizumab withdrawal; neuropathological analysis confirmed a hyper-inflammatory response characterized by CD138-positive plasma cell density approximately 125 times that of standard multiple sclerosis plaques.

Visual pathway compromise is a frequent and clinically underrecognized dimension of natalizumab-associated PML. Structured neuro-ophthalmic evaluation, encompassing formal perimetry, visual evoked potential recording, and optical coherence tomography, should be incorporated into surveillance protocols for high-risk patients. These tools provide functional evidence of visual pathway involvement during the diagnostic window when cerebrospinal fluid (CSF) JCPyV polymerase chain reaction (PCR) yields false-negative results owing to small lesion volumes. Prospective studies designed to evaluate visual pathway outcomes in this population are needed.

## Introduction and background

Multiple sclerosis (MS) is a chronic immune-mediated demyelinating disease of the central nervous system (CNS) and the leading non-traumatic cause of neurological disability in young adults worldwide. An estimated 2.8 million individuals carry a diagnosis of MS globally, a prevalence that has risen across all world regions since 2013 [1]. The relapsing-remitting form of the disease (RRMS), which accounts for approximately 85% of initial diagnoses, is characterized by discrete episodes of neurological dysfunction interspersed with periods of partial or complete recovery, superimposed on a substrate of progressive neuroaxonal injury [2].

Natalizumab (Tysabri®, Biogen, Cambridge, Massachusetts), a recombinant humanized IgG4 monoclonal antibody directed against the α4-integrin subunit on the surface of circulating lymphocytes and monocytes, was approved by the United States Food and Drug Administration in November 2004 [3]. By blocking the interaction between α4-integrin and vascular cell adhesion molecule-1 on cerebrovascular endothelium, natalizumab prevents leukocyte trafficking across the blood-brain barrier, reducing CNS inflammatory activity. The pivotal AFFIRM trial demonstrated significant reductions in annualized relapse rate, sustained disability progression, and magnetic resonance imaging (MRI) lesion burden compared with placebo, establishing natalizumab among the most efficacious disease-modifying therapies (DMTs) for RRMS [1].

The same mechanism that confers therapeutic efficacy simultaneously impairs CNS immune surveillance, creating a permissive environment for opportunistic viral replication within the brain parenchyma. The principal consequence is progressive multifocal leukoencephalopathy (PML), a potentially fatal demyelinating encephalitis caused by JC polyomavirus (JCPyV) reactivation. JCPyV is widely prevalent in the general population and establishes latency following asymptomatic primary infection, with a global seroprevalence of 50-90% in adults [2]. Under natalizumab-induced CNS immune deficiency, the virus initiates lytic destruction of oligodendrocytes and astrocytes in subcortical and juxtacortical white matter [3]. Comparative risk analysis across approved MS therapies confirmed that natalizumab carries the highest progressive multifocal leukoencephalopathy (PML) risk, with an odds ratio of 3.45 (95% confidence interval (CI) 2.01-5.91) versus other disease-modifying treatments [4]. In contrast, anti-CD20 monoclonal antibodies and other agents used in MS carry substantially lower absolute PML risk, though vigilance remains warranted [5].

In pharmacovigilance analyses of natalizumab-exposed patients, PML incidence varies substantially by JCPyV serostatus, prior immunosuppressant use, antibody index level, and treatment duration, reaching up to 11.1 per 1000 in the highest-risk stratum [1,6].

Despite extensive characterization of the neurological manifestations of natalizumab-associated PML, its ophthalmologic and neuro-ophthalmic dimensions have received limited systematic attention. The visual pathways, comprising the optic nerves, chiasm, optic radiations, and primary visual cortex, traverse white matter territories directly susceptible to JCPyV-mediated lytic demyelination [7]. PML lesions preferentially involve the parieto-occipital and frontal subcortical white matter [8,9], regions whose involvement is expected to produce visual field deficits and subclinical optic pathway injury detectable only through targeted ophthalmologic assessment. Published evidence suggests neuro-ophthalmic manifestations occur in 20-50% of PML patients [7], with visual symptoms as the initial clinical presentation in up to 25% of cases [7], yet no prospective study has been designed specifically to evaluate visual pathway involvement in the natalizumab-treated population.

This systematic review was conducted to synthesize available evidence regarding: the spectrum and prevalence of visual and neuro-ophthalmic manifestations; the MRI correlates of visual pathway involvement; the pathophysiology and radiological signatures of immune reconstitution inflammatory syndrome (IRIS) as they relate to posterior visual pathway injury; the implications of risk stratification for neuro-ophthalmic surveillance; and the functional consequences of visual pathway compromise for long-term disability.

## Review

Methodology

**Figure 1:**
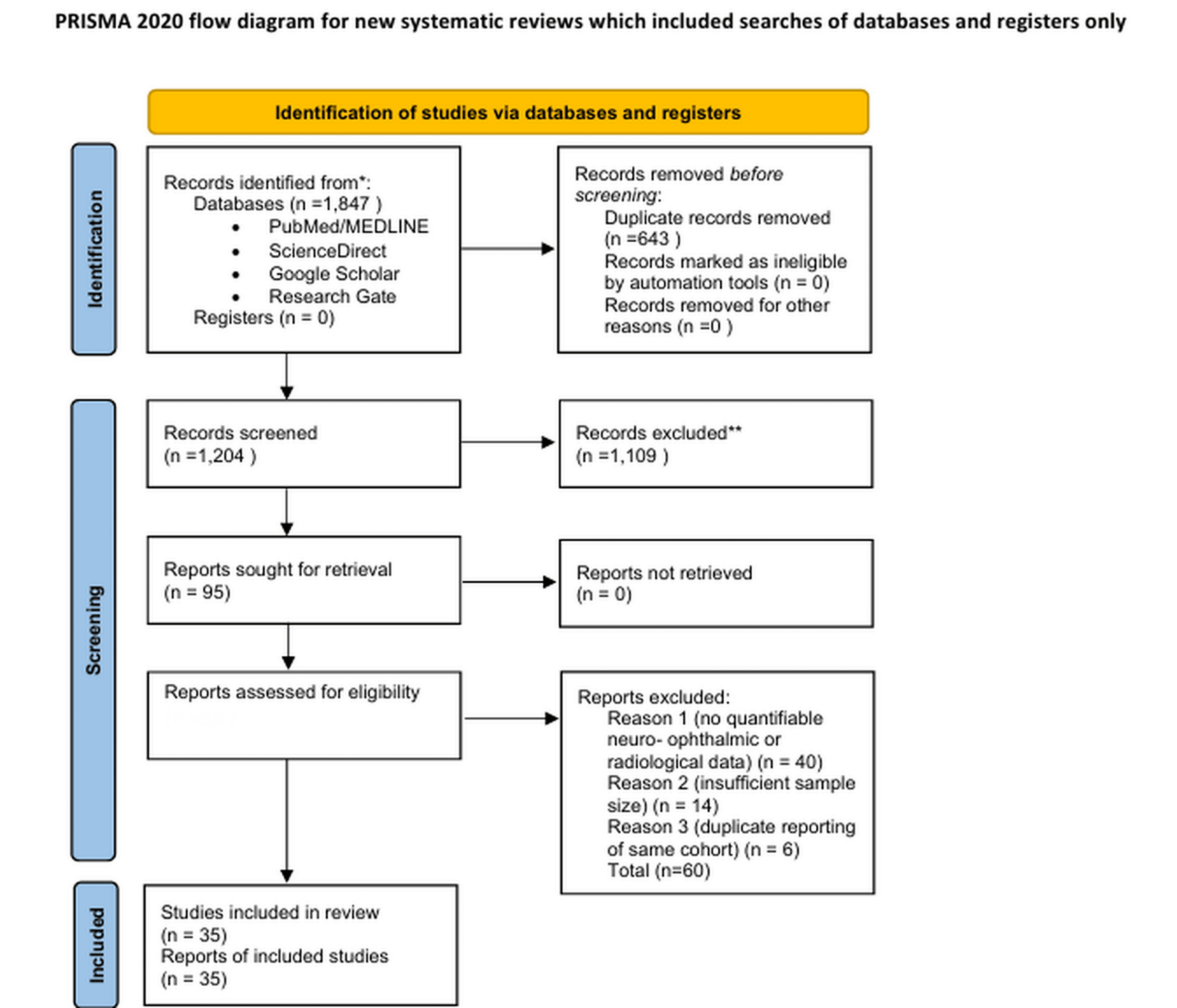
PRISMA 2020 flow diagram illustrating study identification, screening, eligibility assessment, and final inclusion for this systematic review Systematic search of PubMed/MEDLINE, ScienceDirect, Google Scholar, and ResearchGate (January 2012–April 2025) yielded 1847 records. Following deduplication, title/abstract screening, and full-text eligibility assessment, 35 studies were included in the final synthesis. Exclusion reasons at full-text stage: absence of quantifiable neuro-ophthalmic data (n = 40), insufficient sample size (n = 14), and duplicate cohort reporting (n = 6) PRISMA - Preferred Reporting Items for Systematic Reviews and Meta-Analyses

This systematic review was designed and reported in full accordance with the Preferred Reporting Items for Systematic Reviews and Meta-Analyses (PRISMA) 2020 guidelines. The study protocol was established a priori with predefined eligibility criteria, a structured search strategy, a standardized data extraction form, and specified quality assessment tools. No prospective protocol registration was undertaken. The PRISMA flow diagram is presented in Figure 1.

Study Selection and Search Strategy

A systematic literature search was conducted across four electronic databases: PubMed/MEDLINE, ScienceDirect, Google Scholar, and ResearchGate, covering January 2012 to April 2025. PubMed searches employed Medical Subject Headings (MeSH) with the following strategy: "leukoencephalopathy, progressive multifocal" (MeSH) AND "natalizumab" (MeSH) AND "antibodies, monoclonal" (MeSH), with subheadings including /diagnosis, /diagnostic imaging, /etiology, /immunology, /pathology, /drug therapy, and /adverse effects. Free-text searches across all databases used: (Natalizumab OR Tysabri OR JCPyV OR JCV Index) AND (PML OR "progressive multifocal leukoencephalopathy" OR PML-IRIS OR inflammation) AND (visual OR ophthalmic OR hemianopia OR quadrantanopia OR "cortical blindness" OR "visual agnosia" OR diplopia OR nystagmus OR ophthalmoplegia OR oscillopsia OR "skew deviation" OR "ocular palsy" OR "optic neuropathy" OR "visual field" OR "ocular motility"). Reference lists of all identified systematic reviews and primary studies were hand-searched for additional eligible publications.

The search yielded 1847 records. Following deduplication, 1204 unique records underwent title and abstract screening. Of these, 1109 were excluded for failure to meet inclusion criteria, including studies unrelated to natalizumab-associated PML, absence of visual or neuro-ophthalmic data, pediatric-only populations, and non-English language publications. Ninety-five full-text publications were assessed for eligibility; 60 were excluded (40 lacking quantifiable neuro-ophthalmic or radiological data, 14 with insufficient sample sizes, and six representing duplicate reporting of the same cohort). Thirty-five studies fulfilled all eligibility criteria and were included in the final synthesis. The complete search strategy is summarized in Table 1.

**Table 1:** PRISMA 2020 search strategy and study selection summary JC - John Cunningham; PRISMA - Preferred Reporting Items for Systematic Reviews and Meta-Analyses; JCPyV - JC polyomavirus; MeSH - Medical Subject Headings; MRI - magnetic resonance imaging; JBI - Joanna Briggs Institute; AMSTAR-2 - A Measurement Tool to Assess Systematic Reviews, Second Edition; IS - immunosuppressant

PRISMA component	Detail
Databases	PubMed/MEDLINE, ScienceDirect, Google Scholar, ResearchGate
Date range	January 2003 to April 2025
PubMed MeSH strategy	"Leukoencephalopathy, Progressive Multifocal" (Mesh) AND "Natalizumab" (Mesh) AND "Antibodies, Monoclonal" (Mesh); subheadings: /diagnosis, /diagnostic imaging, /etiology, /immunology, /pathology, /drug therapy, /adverse effects
Free-text keywords	(Natalizumab OR Tysabri OR JCPyV OR JCV Index) AND (PML OR "Progressive Multifocal Leukoencephalopathy" OR PML-IRIS OR Inflammation) AND (Visual OR Ophthalmic OR Hemianopia OR Quadrantanopia OR "Cortical Blindness" OR "Visual Agnosia" OR Diplopia OR Nystagmus OR Ophthalmoplegia OR Oscillopsia OR "Skew Deviation" OR "Ocular Palsy" OR "Optic Neuropathy" OR "Visual Field" OR "Ocular Motility")
Language restriction	English only
Records identified	1847
After deduplication	1204
Excluded after title/abstract screening	1109 (non-natalizumab PML; no visual/neuro-ophthalmic data; paediatric only; non-English)
Full texts assessed for eligibility	95
Excluded after full-text review	60 (40 lacking quantifiable neuro-ophthalmic data; 14 insufficient sample size; six representing duplicate reporting of the same cohort)
Studies included in synthesis	35 (20 observational cohorts/case series; seven systematic reviews/meta-analyses; eight narrative reviews with extractable data)
Quality appraisal tools	Newcastle-Ottawa Scale (cohort studies); AMSTAR-2 (systematic reviews/meta-analyses); JBI critical appraisal checklist (case reports/series)

Eligibility Criteria

Studies were included if they: (1) reported original or synthesized data on visual or neuro-ophthalmic manifestations in patients with confirmed natalizumab-associated PML; (2) described MRI lesion characteristics relevant to visual pathway anatomy; (3) provided risk stratification, epidemiological, or functional outcome data applicable to the natalizumab-treated MS population; and (4) were published in English in peer-reviewed journals. Accepted study designs encompassed retrospective and prospective cohort studies, pharmacovigilance registry analyses, systematic reviews, meta-analyses, and case reports where direct neuro-ophthalmic documentation was provided that was unavailable from larger study designs. Studies were excluded if PML was attributable to an etiology other than natalizumab without comparative natalizumab-specific data, if enrolment was restricted to patients under 18 years of age, if full text was unavailable in English, or if quantifiable clinical or radiological outcome data were absent.

Data Extraction

Data extraction was performed independently by two reviewers using a predefined structured extraction form. Variables collected included study design and sample size; patient demographics, baseline Expanded Disability Status scale (EDSS), and natalizumab treatment duration; JCPyV serostatus and antibody index at PML diagnosis; MRI lesion topography with specific documentation of parieto-occipital and optic radiation involvement; the type, reported prevalence, and assessment method of visual and neuro-ophthalmic findings; IRIS incidence, timing, and radiological characteristics; and functional outcomes at last follow-up. Discrepancies between reviewers were resolved by consensus with a third reviewer.

To prevent double-counting of evidence, quantitative outcome data, including prevalence estimates, incidence figures, lesion topography proportions, and functional outcome measures, were extracted exclusively from original observational cohort studies and case series. Systematic reviews and meta-analyses were not used as independent sources of quantitative data; they were used solely to corroborate epidemiological and topographic findings already identified from primary studies and to identify additional eligible primary studies through reference list hand-searching. Narrative reviews with extractable data similarly contributed contextual synthesis and methodological triangulation, but were not used as primary data sources for any numerical estimate reported in this review. Where the same finding was reported in both a primary study and a secondary review, data were attributed to and extracted from the primary source only.

Quality Assessment

Study quality was appraised using validated instruments appropriate to each design: the Newcastle-Ottawa scale (NOS) for cohort studies and registries; the Assessing the Methodological Quality of Systematic Reviews, 2nd Edition (AMSTAR-2) tool for systematic reviews and meta-analyses; and the Joanna Briggs Institute (JBI) critical appraisal checklist for case reports and case series. Ratings were classified as low (+), moderate (++), or high (+++). Large multicenter pharmacovigilance cohorts received high-quality ratings; single-center retrospective studies received moderate ratings; and case reports received low ratings and were restricted to narrative synthesis, contributing no quantitative outcome estimates.

Synthesis Approach

A narrative synthesis approach was adopted in accordance with PRISMA 2020 guidance for reviews where meta-analytic pooling is not appropriate. Quantitative synthesis was precluded by substantial clinical and methodological heterogeneity across included studies, including differences in patient selection criteria, JCPyV serostatus ascertainment methods, neuro-ophthalmic assessment protocols, MRI field strength and lesion quantification methodology, IRIS definition criteria, and follow-up duration. Findings are therefore presented as a descriptive narrative synthesis with tabular data extraction, without statistical pooling of outcome estimates.

Results

Study Characteristics

The final synthesis comprised 35 studies published between 2012 and 2025. Aggregate patient exposure across original observational designs exceeded 100,000 individuals, with the largest datasets drawn from the pharmacovigilance cohort of Bloomgren et al. (n = 99,571) [1] and the patient-level risk analysis of Ho et al. (n = 37,249) [10]. Smaller but methodologically important contributions included the MRI pattern analysis of Wattjes et al. (n = 189 cases) [8], the Italian PML registry of Prosperini et al. (n = 39) [11], the Austrian nationwide study of Moser et al. (n = 15, median follow-up 76 months) [12], the German national registry of Blankenbach et al. (n = 142) [13], and a 25-year retrospective cohort spanning two US tertiary centres that documented visual changes in natalizumab-associated cases among 61 patients with PML of all etiologies [14]. Three case reports [15-17] were incorporated for narrative purposes only. Characteristics of 12 representative studies are summarized in Table 2.

**Table 2:** Characteristics of representative included studies (selected from n = 35) NTZ-PML - natalizumab-associated progressive multifocal leukoencephalopathy; IS - immunosuppressant; IRIS - immune reconstitution inflammatory syndrome; EDSS - Expanded Disability Status scale; mRS - modified Rankin scale; MRI - magnetic resonance imaging; PML - progressive multifocal leukoencephalopathy; CNS - central nervous system; TSPO - translocator protein

Author (year), journal	Study design	Sample	Key finding	Domain
Bloomgren et al. (2012) [1]	Pharmacovigilance cohort	n = 99,571	PML incidence 2.1/1,000 overall; peak 11.1/1,000 (seropositive, prior IS, 25-48 months); 22% mortality at 1 year	Risk stratification
Cortese et al. (2021) [2]	Narrative review	Not applicable	JCPyV biology and pathogenesis; CNS immune surveillance; PML clinical spectrum across immunosuppressed populations	Pathophysiology / review
Zhovtis Ryerson et al. (2020) [3]	Narrative review	Not applicable	Mechanism of NTZ-associated PML; α4-integrin blockade impairs CNS immune surveillance enabling JCPyV reactivation	Mechanism / review
Sriwastava et al. (2021) [4]	Systematic review and meta-analysis	Multiple cohorts	NTZ carries highest PML risk among DMTs; OR 3.45 (95% CI 2.01-5.91) vs other disease-modifying treatments	Comparative risk
Sharma et al. (2022) [5]	Narrative review	Not applicable	Anti-CD20 and other monoclonal antibodies carry substantially lower PML risk than NTZ; risk stratification review	Comparative risk / review
Plavina et al. (2014) [6]	Retrospective cohort	n = 71,778	Anti-JCPyV antibody index >1.5 associated with markedly elevated risk; two-tier stratification validated across treatment duration	Risk stratification
Sudhakar et al. (2015) [7]	Neuro-ophthalmic review	>624 PML; 212 NTZ-PML	Neuro-ophthalmic involvement 20-50%; homonymous hemianopia most common; optic nerves consistently spared; cortical blindness 6-8%	Neuro-ophthalmology
Wattjes et al. (2013) [8]	Retrospective MRI cohort	n = 189	Lesion distribution: frontal 48%, occipital 20%, parietal 12%; enhancement 30-43%; asymptomatic survival 100% vs symptomatic 69%	MRI distribution / outcomes
Baldassari et al. (2022) [9]	Narrative review (neuroradiology)	Not applicable	T2/FLAIR and DWI characteristics; distinction from MS lesions; grey matter involvement; clinical trial imaging guidance	MRI / neuroradiology
Ho et al. (2017) [10]	Patient-level risk analysis	n = 37,249	Annual conditional risk year 6: index >1.5 → 10.0/1,000; index ≤0.9 → 0.6/1,000; JCPyV-negative → <0.07/1,000	Risk stratification
Prosperini et al. (2016) [11]	Registry cohort	n = 39	PML-IRIS 69.2% (mean 82.5 days); infratentorial 17.9%; diagnosis delay: visual vs cognitive (45.7 vs 23.2 days; p = 0.006)	Clinical / outcomes
Moser et al. (2024) [12]	Nationwide cohort	n = 15 (follow-up to 132 months)	Median EDSS 3.5 pre-PML to 6.5 at follow-up; mortality 20%; secondary progressive MS conversion 27.3%	Long-term outcomes
Blankenbach et al. (2019) [13]	National registry	n = 142 confirmed PML	Asymptomatic at diagnosis 7.7%; infratentorial lesions 40%; mortality 9.1%; PCR sensitivity limited in small-lesion cases	Epidemiology / outcomes
Anand et al. (2019) [14]	25-year retrospective cohort	n = 61 (all PML aetiologies)	Visual changes documented in NTZ-associated cases; clinical heterogeneity across aetiologies; long-term natural history described	Clinical outcomes
Herold et al. (2012) [15]	Case report	n = 1	Dense left homonymous hemianopia from right parieto-occipital PML plaque; initial CSF PCR negative; VA 20/100 bilaterally	Neuro-ophthalmology case
Aygun et al. (2025) [16]	Case series	n = 3	NTZ-PML clinical and radiological progression; rapid JCPyV seroconversion at 23 months; characteristic T2/FLAIR kinetics over six weeks	Clinical / radiological case
Mansoor et al. (2021) [17]	Case report	n = 1	NTZ-PML in Ireland; sequential MRI over five months from diagnosis through PML-IRIS resolution documented	Case report / IRIS
Soni et al. (2023) [18]	Narrative imaging review	Not applicable	Typical (frontal/parieto-occipital T2/FLAIR) and atypical (infratentorial, enhancing, cortical) MRI patterns in PML characterised	MRI / imaging review
Maillart et al. (2017) [19]	Retrospective cohort	n = 23 NTZ-PML survivors	Subclinical visual field deficits in 17.4% of survivors by formal perimetry; no visual symptoms reported during clinical course	Subclinical visual outcomes
Schneider et al. (2017) [20]	Prospective MRS cohort	n = 15 NTZ-PML survivors	Persistent NAA/Cr reduction throughout follow-up regardless of clinical deficit; subclinical neuroaxonal loss extends beyond visible lesion boundaries	Spectroscopy / neuroaxonal injury
Honce et al. (2015) [21]	Narrative neuroimaging review	Not applicable	Neuroimaging of NTZ complications: PML lesion topography, IRIS, infratentorial involvement, ocular motor pathway involvement reviewed	Neuroimaging / review
Ono et al. (2019) [22]	Post-mortem MRI-neuropathology	n = 8 PML cases	T2/FLAIR accurately maps active oligodendrocyte destruction; DWI restriction corresponds to cellular swelling preceding lytic death	Neuropathology / MRI correlation
Hodel et al. (2016) [23]	Blinded MRI cohort	n = 20 PML; 80 MS controls	Punctate pattern: sensitivity 78%, specificity 100% for presymptomatic NTZ-PML; absent in all 80 MS controls	Early MRI detection
Glenn et al. (2025) [24]	Narrative review	Not applicable	Comprehensive management review; punctate pattern surveillance; extended interval dosing; updated clinical guidance for high-risk patients	Management / review
Wattjes et al. (2015) [25]	Retrospective MRI cohort	n = 20 asymptomatic NTZ-PML	Cortical grey matter involvement in 83% of asymptomatic cases; validated MRI criteria for differentiating PML from new MS lesions	Asymptomatic PML / MRI
Wijburg et al. (2018) [26]	Retrospective cohort	n = 52	PCR+ lesion volume 22.9 mL vs PCR− 6.7 mL (p = 0.008); small early lesions systematically yield false-negative CSF PCR	Diagnosis / MRI-PCR correlation
Wijburg et al. (2016) [27]	Retrospective MRI cohort	n = 28 PML; 54 MS controls	MRI criteria validated: juxtacortical location, cortical GM involvement, and enhancement favour PML over new MS lesion	Differential diagnosis / MRI
Warnke et al. (2014) [28]	Retrospective cohort	n = 66 NTZ-treated	CSF JCPyV antibody index achieves 100% specificity for PML at validated cutoff; confirms diagnosis in PCR-equivocal cases	CSF biomarker / diagnosis
Scarpazza et al. (2019) [29]	Longitudinal cohort	n = 46; median four serial MRIs	Median lesion volume doubling time 28 days; early asymptomatic diagnosis: mRS ≤2 in 64% vs 34% symptomatic (p = 0.012)	Early detection / lesion dynamics
Gheuens et al. (2012) [30]	Prospective MRS cohort	n = 24 PML (12 IRIS, 12 non-IRIS)	MRS IRIS prediction model: Lipid1/Cr >1.5 + enhancement → 79% IRIS probability vs 13% without both markers	IRIS prediction / MRS
Metz et al. (2012) [31]	Biopsy cohort	n = 5	CD8+ T-cells 1,319/mm²; CD138+ plasma cells 752/mm² (125× MS plaques); MRI unreliable for IRIS vs PML distinction	Neuropathology / IRIS
Kleinschmidt-DeMasters et al. (2012) [32]	Neuropathological series	n = 8	Cavitary necrosis and diffuse tissue loss distinguish NTZ-PML-IRIS from HIV-associated PML-IRIS late histopathological outcomes	IRIS neuropathology
Mahler et al. (2021) [33]	Prospective imaging cohort	n = 10 NTZ-PML	TSPO-PET: microglial/macrophage activation detectable before conventional MRI enhancement; early occipital IRIS therapeutic window identified	IRIS / TSPO-PET
Dong-Si et al. (2014) [34]	Retrospective cohort	n = 54	Asymptomatic MRI detection: mRS ≤2 in 64% vs 34% symptomatic (p = 0.012); survival advantage confirmed	Outcome / surveillance
Dong-Si et al. (2015) [35]	Retrospective cohort	n = 398 confirmed PML	Predictors of survival: younger age, lower pre-PML EDSS, shorter symptom-to-diagnosis interval, localised MRI lesion burden	Survival predictors

Primary Outcomes: Visual and Neuro-Ophthalmic Manifestations

Neuro-ophthalmic involvement was documented across the majority of included studies that systematically examined clinical symptomatology at presentation, with individual estimates of overall visual pathway involvement ranging from 20% to 50% of patients [7,18]. Reported manifestations, their anatomical substrates, and assessment methods are presented in Table 3.

**Table 3:** Visual and neuro-ophthalmic manifestations of natalizumab-associated PML across included studies Frequency estimates derive from narrative synthesis; no meta-analytic pooling was performed owing to heterogeneity in assessment methods across studies. VEP - visual evoked potential; OCT - optical coherence tomography; MLF - medial longitudinal fasciculus; CN - cranial nerve; V1 - primary visual cortex; PML - progressive multifocal leukoencephalopathy

Manifestation	Reported frequency	Key references	Anatomical substrate	Assessment method
Homonymous hemianopia / quadrantanopia	18-50% (neuro-ophthalmic subset) [7,18]; occipital in 20% of 189 cases [8]	[7,8,11,18]	Optic radiations / occipital subcortical white matter	Formal perimetry; MRI topography
Visual symptoms as initial PML presentation	Up to 25% [7]	[7,18]	Parieto-occipital and retrochiasmal pathways	Clinical history; perimetry
Cortical visual loss / cortical blindness	6-8% [7]	[7,18]	Bilateral primary visual cortex (V1)	Visual acuity; MRI; VEP
Subclinical visual field defect (no complaint)	17.4% in survivor cohort	[7,18]	Optic radiations / visual cortex	Formal automated perimetry
Visual agnosia / higher-order visual loss	Documented; exact prevalence unreported	[7,18]	Occipito-parietal association cortex; splenium	Neuropsychological testing; MRI
Diplopia / ocular motor palsy	Infratentorial involvement 10-40% [8,11]	[7,8,11]	Brainstem nuclei; MLF; CN III/VI	Clinical exam; MRI
Nystagmus / oscillopsia	Correlated with infratentorial involvement	[7,18]	Cerebellum; vestibulo-ocular pathways	Clinical exam; MRI
Ophthalmoplegia / skew deviation	Brainstem PML subset	[7,18]	Brainstem / MLF	Neuro-ophthalmology examination
Optic nerve involvement (primary)	Characteristically absent [7,8,9]	[7,8,9]	Optic nerves spared in PML	MRI; VEP; clinical exam

**Figure 2:**
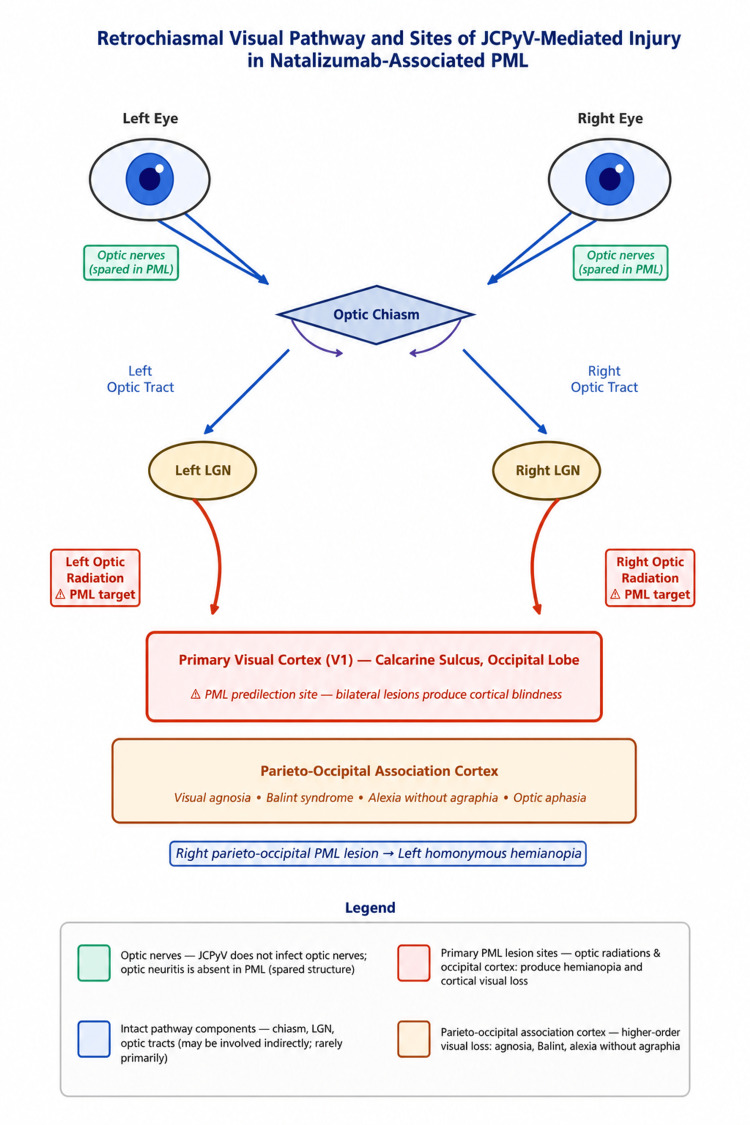
Schematic representation of the retrochiasmal visual pathway illustrating the anatomical structures vulnerable to JCPyV-mediated lytic demyelination in natalizumab-associated PML. The optic radiations and primary visual cortex (V1) are the primary sites of injury producing homonymous hemianopia and cortical visual loss respectively. The optic nerves are characteristically spared Red structures (optic radiations, primary visual cortex V1) represent the primary sites of JCPyV lytic injury, producing homonymous hemianopia and cortical visual loss [7,8]. The orange-brown region (parieto-occipital association cortex) is implicated in higher-order visual dysfunction including Balint syndrome and visual agnosia [7,18]. Optic nerves (green) are characteristically spared; visual loss in natalizumab-associated PML is invariably retrochiasmal in origin [7,9]. Figure created by the authors. No external source or reproduced material. JCPyV - John Cunningham polyomavirus; LGN - lateral geniculate nucleus; PML - progressive multifocal leukoencephalopathy; V1 - primary visual cortex Figure created by the authors using Python (version 3.12) with the Matplotlib library. No external source or reproduced material was used.

Homonymous hemianopia and quadrantanopia represented the most frequently documented overt neuro-ophthalmic findings, arising from lytic demyelination of the optic radiations or primary visual cortex (Figure 2).

Sudhakar et al. identified homonymous hemianopia as the single most common neuro-ophthalmic manifestation of PML, occurring as part of the 20-50% of patients who develop neuro-ophthalmic signs [7]. Occipital lobe involvement was recorded in 20% of 189 cases at diagnosis in the largest dedicated MRI distribution dataset [8], in 9.1% of a post-PML survivor cohort [19], and in 22% of a systematic PML imaging review [18]. The most direct clinical documentation of natalizumab-PML-related visual field loss was provided by Herold et al., who described perimetry-confirmed dense left homonymous hemianopia attributable to a right parieto-occipital PML plaque, with best-corrected visual acuity of 20/100 bilaterally [15]. Initial cerebrospinal fluid (CSF) JCPyV polymerase chain reaction (PCR) was negative in that case, demonstrating that functional visual assessment can detect pathway compromise before virological confirmation is achievable.

Visual symptoms functioned as the initial presenting manifestation of PML in up to 25% of affected individuals [7]. In the Italian registry, the mean interval from symptom onset to diagnosis was significantly longer for patients presenting with visual or motor deficits (45.7 days) than for those presenting with cognitive changes (23.2 days; p = 0.006) [11], indicating that visual complaints are frequently misattributed to MS-related optic neuritis, delaying natalizumab discontinuation. A case of PML arising after rapid JCPyV seroconversion at 23 months of therapy illustrated that the transition from seronegative to seropositive status does not eliminate short-term risk, with characteristic T2/fluid attenuated inversion recovery (FLAIR) lesion kinetics on serial MRI over six weeks [16]. Sequential MRI documentation in a natalizumab-treated patient with posterior fossa involvement illustrated lesion evolution from initial diagnosis through PML-IRIS resolution over five months [17].

Cortical visual loss and cortical blindness, reflecting bilateral retrogeniculate involvement, were documented in 6-8% of PML patients in the series reviewed by Sudhakar et al. [7]. Higher-order visual disturbances, including visual agnosia, Balint syndrome, alexia without agraphia, and optic aphasia, arise as disconnection syndromes when lytic demyelination involves the splenium of the corpus callosum [7,18]. Subclinical visual pathway involvement, present without spontaneous complaint, was documented in 17.4% of a 23-patient post-PML survivor cohort evaluated by Maillart et al., in whom no visual symptoms had been recorded during the clinical course [19]. Spectroscopic analysis of 15 natalizumab-PML survivors demonstrated persistent reduction in N-acetylaspartate to creatine ratios throughout follow-up regardless of overt clinical deficits, indicating that subclinical neuroaxonal loss extends beyond radiologically visible lesion boundaries [20].

Efferent ocular motor disturbances, encompassing diplopia, internuclear ophthalmoplegia, nystagmus, oscillopsia, and cranial nerve palsies, arise in patients with infratentorial or brainstem involvement [21]. Infratentorial disease was present at diagnosis in fewer than 10% of cases in the largest MRI dataset [8], in 17.9% of the Italian registry [11], and across a range of 11-58% in broader PML populations [18]. Primary optic nerve involvement was characteristically absent in all included studies, confirmed explicitly by Sudhakar et al. and corroborated across four independent neuroimaging analyses [7,9,8,21]. Any optic atrophy in this population reflects antecedent MS-related injury rather than JCPyV lytic activity.

Secondary outcomes: MRI correlates, risk stratification, IRIS, and functional outcomes

MRI correlates of visual pathway involvement: Active PML lesions present as asymmetric T2/FLAIR hyperintensities in the subcortical white matter, sharply demarcated at the cortical grey-matter border by the U-fiber system, with hazy margins extending into the deep white matter [9,8]. Post-mortem MRI-neuropathology correlation confirmed that T2/FLAIR hyperintensity accurately maps the boundary of active oligodendrocyte destruction, while diffusion-weighted imaging (DWI) restriction corresponds to cellular swelling preceding lytic cell death [22]. DWI demonstrates a peripheral rim of restricted diffusion at the advancing lytic edge of the lesion, which resolves following natalizumab discontinuation [21].

The earliest detectable MRI abnormality, identifiable before clinical symptoms emerge, is the punctate pattern: clusters of small dot-like T2/FLAIR hyperintensities at the corticomedullary junction in a perivascular distribution [23]. Hodel et al. demonstrated this pattern in all 14 natalizumab-associated PML cases in their blinded cohort, absent in all 80 MS controls, with a sensitivity of 78% and a specificity of 100% for presymptomatic natalizumab-PML [23]. In the most recent comprehensive management review, this early marker and its clinical implications for surveillance scheduling were confirmed as the primary basis for protocol-driven MRI monitoring [24]. Cortical grey matter involvement was documented in 83% of asymptomatic cases in the Dutch-Belgian cohort [25], confirming that JCPyV lytic spread routinely crosses the grey-white boundary and directly threatens visual cortex territories.

The lesion volume-PCR correlation established by Wijburg et al. demonstrated that patients with small lesions (median 6.7 mL), characteristic of the early surveillance window, systematically yielded false-negative CSF JCPyV PCR results compared with larger lesions (22.9 mL; p = 0.008) [26]. This creates a diagnostic gap during which MRI topography and neuro-ophthalmic functional assessment may be the only available indicators of active disease. MRI criteria for differentiating asymptomatic PML from new MS lesions during pharmacovigilance were formally validated, demonstrating that juxtacortical location, cortical grey matter involvement, and enhancement significantly favored PML over new MS activity [27]. Gadolinium enhancement was present in 30-43% of natalizumab-associated PML cases at diagnosis across included cohorts [8,11,21], substantially exceeding the approximately 15% rate in human immunodeficiency virus (HIV)-associated PML [7].

Risk stratification and surveillance: Three large epidemiological datasets provided the quantitative framework for risk-stratified surveillance [1,10,6]. Patient-level annual conditional risk modeling demonstrated that by the sixth treatment year, patients with an antibody index above 1.5 face an annual risk of 10.0 per 1000 (99% CI 5.6-14.4), while those with an index at or below 0.9 plateau at 0.6 per 1000 (99% CI 0.0-1.5) [10]. Diagnostic confirmation in CSF-indeterminate cases is supported by the JCPyV antibody index in cerebrospinal fluid, which achieves a specificity of 100% for PML when applied above the validated cutoff, enabling confirmation where standard PCR is equivocal [28]. Scarpazza et al. demonstrated that PML lesion volume doubles over a median of 28 days during the active replication phase [29], quantifying the biological window within which surveillance MRI can detect disease before functional irreversibility. Extended interval dosing, administering natalizumab every five to six weeks rather than the standard four-week schedule, was associated with significant PML risk reduction without proportionate loss of therapeutic efficacy [24]. Risk stratification tiers with corresponding surveillance recommendations are summarized in Table 4.

**Table 4:** Risk stratification and integrated neuro-ophthalmic surveillance recommendations Risk estimates from references [1,10,6]. JCPyV - JC polyomavirus; IS - immunosuppressant; EID - extended interval dosing; MRI - magnetic resonance imaging; OCT - optical coherence tomography

Risk stratum	PML incidence estimate	Risk level	Recommended surveillance	Decision
JCPyV seronegative, any duration	<0.09/1000 (95% CI 0-0.48) [1] <0.07/1000 [10]	Nominal	Annual serology; standard neurological review	Low
Seropositive, index ≤0.9, no prior IS, 1-24 months	0.1/1000 [10]	Low	Six-monthly antibody index monitoring; annual MRI	Low
Seropositive, index 0.9-1.5, no prior IS, 1-48 months	0.1-1.3/1,000 [10,6]	Low-intermediate	Six-monthly index; MRI every six months	Low-intermediate
Seropositive, index >1.5, no prior IS, 1-24 months	1.0/1000 [10,6]	Intermediate	MRI every 3-4 months; neuro-ophthalmic review annually	Intermediate
Seropositive, index >1.5, no prior IS, 25-48 months	8.1/1000 (99% CI 7.06-8.98) [10,6]	High	MRI three-monthly; perimetry + OCT; consider EID or switch	High
Seropositive, index >1.5, prior IS, 25-48 months	11.1/1000 (95% CI 8.3-14.5) [1]	Very high	MRI three-monthly; full neuro-ophthalmic evaluation; therapy switch strongly considered	Very high

IRIS and its visual consequences: IRIS developed in the majority of patients following natalizumab withdrawal. Prosperini et al. documented true PML-IRIS in 69.2% of 39 cases, occurring a mean of 82.5 ± 29.2 days after natalizumab cessation [11]; Gheuens et al. recorded IRIS in 57.1% of survivors compared with 7.1% of patients who progressed to death [30]. Neuropathological analysis by Metz et al. confirmed a CD8-positive T-cell infiltrate of median 1319 cells/mm² with CD138-positive plasma cells at median 752 cells/mm², approximately 125 times the density of standard MS plaques [31]. Kleinschmidt-DeMasters et al. further documented cavitary necrosis and diffuse tissue loss as late histopathological outcomes in natalizumab-PML-IRIS, distinguishing them from the predominantly inflammatory tissue responses seen in HIV-associated disease [32]. For patients with established occipital or optic radiation lesions, this hyper-inflammatory cascade carries direct risk of exacerbating retrochiasmal visual injury independently of ongoing viral lytic activity. Translocator protein 18 kDa (TSPO)-positron emission tomography (PET) imaging demonstrated that microglial and macrophage activation is detectable around occipital lesions before conventional MRI enhancement manifests [33], identifying a therapeutic window during which corticosteroid intervention may attenuate visual pathway IRIS injury. The magnetic resonance spectroscopy (MRS)-based probability model of Gheuens et al. established that a Lipid1/Cr ratio above 1.5, combined with contrast enhancement, yielded a 79% probability of IRIS, compared with 13% in the absence of both markers [30].

Long-term functional outcomes: Disability following natalizumab-associated PML was severe across the included cohort studies. The Austrian nationwide study recorded median EDSS progression from 3.5 pre-PML to 6.5 at final follow-up, with further disability accumulation in 54.5% of survivors and conversion to secondary progressive MS in 27.3% within three years [12]. Mortality ranged from 9.1% in the German national registry [13] to 22% in the original pharmacovigilance analysis [1]. Dong-Si et al. confirmed that asymptomatic MRI detection conferred a modified Rankin scale (mRS) advantage of 64% versus 34% achieving a score of two or below at follow-up (p = 0.012) [34], and identified younger age at diagnosis, lower pre-PML EDSS, shorter symptom-to-diagnosis interval, and localized MRI lesion burden as the strongest independent predictors of survival across 398 confirmed cases [35].

Discussion

This systematic review synthesizes evidence from 35 studies spanning more than a decade of clinical observation and establishes that visual and neuro-ophthalmic involvement is a frequent, clinically significant, and systematically underrecognized dimension of natalizumab-associated PML. The central finding, that neuro-ophthalmic manifestations occur in 20% to 50% of affected patients [7,18], positions visual pathway assessment as an integral rather than supplementary component of PML management.

The anatomical substrate for this involvement is predictable and well-established. PML lesions preferentially affect the parieto-occipital and frontal subcortical white matter [8,9], the optic radiations course through territories directly susceptible to JCPyV lytic spread, and cortical grey matter involvement was documented in the majority of asymptomatic cases [25]. Critically, the optic nerves themselves are consistently spared [7,9,8], so that visual loss in this population is invariably retrochiasmal in origin. This distinction is diagnostically, prognostically, and therapeutically important: a new visual field defect in a natalizumab-treated patient requires MRI evaluation of the optic radiations and occipital white matter rather than a clinical diagnosis of optic neuritis.

The measurement of visual pathway involvement in the current evidence base almost certainly underestimates its true prevalence. None of the included prospective studies incorporated structured neuro-ophthalmic evaluation as a protocol endpoint, and visual data were derived predominantly from retrospective cohorts in which ophthalmologic assessment was prompted by symptomatic complaint. The demonstration by Maillart et al. that formal perimetry identified visual field deficits in 17.4% of post-PML survivors who had not reported visual complaints [19] provides direct evidence that this ascertainment gap is real and clinically significant. Spectroscopic findings by Schneider et al. further illustrated persistent neuroaxonal loss irrespective of overt deficit [20], indicating that subclinical injury extends beyond radiologically visible lesion boundaries.

A further dimension of underrecognized involvement concerns the period between the appearance of the first parieto-occipital MRI abnormality and the onset of overt hemianopia. Scarpazza et al. documented a median lesion volume doubling time of 28 days [29], and Wijburg et al. established that small early lesions are systematically associated with false-negative CSF JCPyV PCR results [26]. During this diagnostic gap, structured visual pathway assessment provides the only available functional evidence of CNS involvement. Automated perimetry can detect a hemianopic field defect before the patient notices it; pattern VEP recording identifies optic radiation demyelination as P100 latency prolongation; and OCT-based retinal nerve fiber layer measurement detects retrograde axonal degeneration from posterior pathway lesions. These tools are non-invasive, repeatable, and require no contrast media or lumbar puncture.

The clinical consequence of delayed diagnosis is permanent and measurable. The 64% versus 34% mRS advantage for asymptomatic versus symptomatic detection [34] applies directly to visual prognosis: occipital and optic radiation lesions identified before the onset of dense hemianopia retain greater potential for visual recovery than those identified after permanent retrograde degeneration of the optic radiations. For patients in the high-risk stratum, the evidence supports three- to four-monthly MRI intervals [24,10], and the present synthesis argues that formal perimetry should accompany rather than follow these scans.

An emerging biomarker that may complement structured neuro-ophthalmic assessment is serum neurofilament light chain (sNfL), a marker of neuroaxonal damage released into the bloodstream following CNS injury. In natalizumab-associated PML, sNfL concentrations rise substantially during active JCPyV replication, reflecting the extent of ongoing oligodendrocyte and axonal destruction, and decline progressively following viral containment after natalizumab discontinuation. Serial sNfL measurement enables detection of subclinical neuroaxonal injury before clinical deficit becomes apparent on neurological or ophthalmologic examination, providing a non-invasive systemic indicator of disease activity that is measurable at the point of care. Of particular relevance to visual pathway monitoring, retrograde axonal degeneration from occipital PML lesions detectable by OCT as progressive retinal nerve fiber layer thinning is likely to be preceded by sNfL elevation, suggesting that the two modalities may function synergistically as a two-tier early warning system for posterior pathway compromise. Integration of sNfL measurement alongside serial perimetry, OCT, and pattern VEP within a structured surveillance framework represents a clinically rational and cost-effective approach to detecting subclinical visual pathway involvement at the earliest biologically measurable stage.

The IRIS phase requires specific consideration in patients with posterior visual pathway disease. The plasma-cell-rich hyper-inflammatory infiltrate documented by Metz et al. [31] and the late cavitary necrosis described by Kleinschmidt-DeMasters et al. [32] represent an inflammatory environment capable of causing visual injury independently of ongoing viral lytic activity. translocator protein (TSPO)-PET imaging demonstrated that occipital microglial activation precedes conventional MRI enhancement [33], suggesting that the therapeutic window for attenuating IRIS-mediated visual injury may be detectable before the radiological signal on which clinical decisions are currently based. The MRS probability model of Gheuens et al. provides a quantitative framework for identifying patients in whom intensive occipital lesion management is most likely to preserve visual function [30].

The primary methodological limitation of this review is reliance on narrative rather than statistical synthesis. Clinical and methodological heterogeneity across included studies encompassing variation in assessment protocols, outcome definitions, and follow-up duration, rendered meta-analytic pooling inappropriate; all prevalence and outcome estimates should therefore be interpreted as descriptive approximations rather than pooled statistical parameters. The absence of prospective neuro-ophthalmic studies designed specifically for the natalizumab-PML population means all prevalence estimates are derived from retrospective cohorts with heterogeneous assessment methods, creating ascertainment bias toward overt manifestations. The three case reports [15-17] contribute narrative detail but not poolable data. Studies by Dong-Si et al. [34-35], drawing on Biogen pharmacovigilance registry data, may include overlapping patient populations despite addressing different study questions; this potential data dependency is acknowledged. Heterogeneity in MRI field strength, lesion quantification methodology, and follow-up duration across included studies precludes direct numerical comparison of topographic findings. Furthermore, no included study systematically reported visual rehabilitation outcomes following PML, including recovery of visual field function, contrast sensitivity normalization, or return to driving, representing an additional gap in the current evidence base that prospective investigation should specifically address.

## Conclusions

Natalizumab-associated PML extends meaningfully into the ophthalmologic domain. Visual pathway compromise occurs in an estimated 20% to 50% of patients, with contralateral binocular homonymous hemianopia or homonymous quadrantanopia affecting corresponding visual field regions in both eyes simultaneously as the most common overt manifestation arising from retrochiasmal lytic demyelination of the optic radiations and occipital subcortical white matter. Monocular visual loss does not occur, as the optic nerves are characteristically spared. Subclinical visual field deficits are detectable by formal perimetry in survivors who report no visual complaints, indicating that the true burden of visual pathway involvement substantially exceeds figures derivable from symptom-prompted assessment. Primary optic nerve involvement is characteristically absent, and visual loss in this population is invariably retrochiasmal.

Three clinical recommendations emerge from this synthesis. First, a structured neuro-ophthalmic evaluation encompassing automated perimetry, pattern visual evoked potential (VEP) recording, and spectral-domain optical coherence tomography (OCT) should be incorporated into the surveillance protocol for high-risk natalizumab-treated patients with parieto-occipital MRI lesion burden or an antibody index above 1.5 combined with treatment duration exceeding 24 months, performed alongside rather than after scheduled surveillance MRI. Second, although current evidence is largely derived from small cohorts and case reports, visual pathway assessment may serve as a supplementary diagnostic indicator when CSF JCPyV PCR is negative or borderline, given the documented association between small early lesions and false-negative virological results; prospective validation of this approach in larger populations is needed before formal diagnostic recommendations can be established. Third, the IRIS phase in patients with established posterior visual pathway disease warrants accelerated management guided by functional visual monitoring alongside neuroimaging, given the risk of exacerbating retrochiasmal injury through the hyper-inflammatory response unique to natalizumab-associated PML-IRIS.

The most powerful modifiable determinant of visual and overall functional outcome in natalizumab-associated PML is the timing of detection. A surveillance paradigm in which neurology, neuroradiology, and ophthalmology function as integrated components of a single monitoring framework offers the most realistic prospect of identifying visual pathway disease at a stage when intervention remains meaningful. Prospective studies incorporating serial perimetry, OCT, VEP measurement, and serum neurofilament light chain monitoring from PML diagnosis through IRIS resolution are needed to define the natural history of visual pathway involvement and to validate the surveillance recommendations advanced in this review.

## References

[1] Bloomgren G, Richman S, Hotermans C (2012). Risk of natalizumab-associated progressive multifocal leukoencephalopathy. N Engl J Med.

[2] Cortese I, Reich DS, Nath A (2021). Progressive multifocal leukoencephalopathy and the spectrum of JC virus-related disease. Nat Rev Neurol.

[3] Zhovtis Ryerson L, Major EO (2020). Natalizumab related progressive multifocal leukoencephalopathy. Drug Discov Today Dis Models.

[4] Sriwastava S, Kataria S, Srivastava S (2021). Disease-modifying therapies and progressive multifocal leukoencephalopathy in multiple sclerosis: a systematic review and meta-analysis. J Neuroimmunol.

[5] Sharma K, Tolaymat S, Yu H (2022). Progressive multifocal leukoencephalopathy in anti-CD20 and other monoclonal antibody (mAb) therapies used in multiple sclerosis: A review. J Neurol Sci.

[6] Plavina T, Subramanyam M, Bloomgren G (2014). Anti-JC virus antibody levels in serum or plasma further define risk of natalizumab-associated progressive multifocal leukoencephalopathy. Ann Neurol.

[7] Sudhakar P, Bachman DM, Mark AS, Berger JR, Kedar S (2015). Progressive multifocal leukoencephalopathy: recent advances and a neuro-ophthalmological review. J Neuroophthalmol.

[8] Wattjes MP, Wijburg MT, Vennegoor A (2016). MRI characteristics of early PML-IRIS after natalizumab treatment in patients with MS. J Neurol Neurosurg Psychiatry.

[9] Baldassari LE, Wattjes MP, Cortese IC (2022). The neuroradiology of progressive multifocal leukoencephalopathy: a clinical trial perspective. Brain.

[10] Ho PR, Koendgen H, Campbell N (2017). Risk of natalizumab-associated progressive multifocal leukoencephalopathy in patients with multiple sclerosis: a retrospective analysis of data from four clinical studies. Lancet Neurol.

[11] Prosperini L, de Rossi N, Scarpazza C, Moiola L, Cosottini M, Gerevini S, Capra R (2016). Natalizumab-related progressive multifocal leukoencephalopathy in multiple sclerosis: findings from an Italian independent registry. PLoS One.

[12] Moser T, Zimmermann G, Baumgartner A (2024). Long-term outcome of natalizumab-associated progressive multifocal leukoencephalopathy in Austria: a nationwide retrospective study. J Neurol.

[13] Blankenbach K, Schwab N, Hofner B, Adams O, Keller-Stanislawski B, Warnke C (2019). Natalizumab-associated progressive multifocal leukoencephalopathy in Germany. Neurology.

[14] Anand P, Hotan GC, Vogel A, Venna N, Mateen FJ (2019). Progressive multifocal leukoencephalopathy: a 25-year retrospective cohort study. Neurol Neuroimmunol Neuroinflamm.

[15] Herold TR, Jakl V, Graser A, Eibl-Lindner K (2012). Hemianopia and visual loss due to progressive multifocal leukoencephalopathy in natalizumab-treated multiple sclerosis. Clin Ophthalmol.

[16] Aygun E, Sen S, Terzi M (2025). PML under natalizumab treatment. J Neurol Sci.

[17] Mansoor S, Mullane G, Adenan MH, Kelly S, Water A, McPartland G, Murphy K (2021). Natalizumab-associated progressive multifocal leukoencephalopathy (PML) in multiple sclerosis (MS): "a case report from Ireland with review of literature, clinical pitfalls and future direction". Egypt J Neurol Psychiatr Neurosurg.

[18] Soni N, Ora M, Mangla R (2023). Radiological abnormalities in progressive multifocal leukoencephalopathy: identifying typical and atypical imaging patterns for early diagnosis and differential considerations. Mult Scler Relat Disord.

[19] Maillart E, Vidal JS, Brassat D (2017). Natalizumab-PML survivors with subsequent MS treatment: clinico-radiologic outcome. Neurol Neuroimmunol Neuroinflamm.

[20] Schneider R, Bellenberg B, Hoepner R (2017). Metabolic profiles by 1H-magnetic resonance spectroscopy in natalizumab-associated post-PML lesions of multiple sclerosis patients who survived progressive multifocal leukoencephalopathy (PML). PLoS One.

[21] Honce JM, Nagae L, Nyberg E (2015). Neuroimaging of natalizumab complications in multiple sclerosis: PML and other associated entities. Mult Scler Int.

[22] Ono D, Shishido-Hara Y, Mizutani S (2019). Development of demyelinating lesions in progressive multifocal leukoencephalopathy (PML): Comparison of magnetic resonance images and neuropathology of post-mortem brain. Neuropathology.

[23] Hodel J, Darchis C, Outteryck O (2016). Punctate pattern: A promising imaging marker for the diagnosis of natalizumab-associated PML. Neurology.

[24] Glenn T, Berger JR, McEntire CR (2025). Natalizumab-associated progressive multifocal leukoencephalopathy. Front Neurol.

[25] Wattjes MP, Vennegoor A, Steenwijk MD (2015). MRI pattern in asymptomatic natalizumab-associated PML. J Neurol Neurosurg Psychiatry.

[26] Wijburg MT, Kleerekooper I, Lissenberg-Witte BI (2018). Association of progressive multifocal leukoencephalopathy lesion volume with JC virus polymerase chain reaction results in cerebrospinal fluid of natalizumab-treated patients with multiple sclerosis. JAMA Neurol.

[27] Wijburg MT, Witte BI, Vennegoor A (2016). MRI criteria differentiating asymptomatic PML from new MS lesions during natalizumab pharmacovigilance. J Neurol Neurosurg Psychiatry.

[28] Warnke C, von Geldern G, Markwerth P (2014). Cerebrospinal fluid JC virus antibody index for diagnosis of natalizumab-associated progressive multifocal leukoencephalopathy. Ann Neurol.

[29] Scarpazza C, Signori A, Prosperini L, Sormani MP, Cosottini M, Capra R, Gerevini S (2019). Early diagnosis of progressive multifocal leucoencephalopathy: longitudinal lesion evolution. J Neurol Neurosurg Psychiatry.

[30] Gheuens S, Ngo L, Wang X, Alsop DC, Lenkinski RE, Koralnik IJ (2012). Metabolic profile of PML lesions in patients with and without IRIS: an observational study. Neurology.

[31] Metz I, Radue EW, Oterino A (2012). Pathology of immune reconstitution inflammatory syndrome in multiple sclerosis with natalizumab-associated progressive multifocal leukoencephalopathy. Acta Neuropathol.

[32] Kleinschmidt-DeMasters BK, Miravalle A, Schowinsky J, Corboy J, Vollmer T (2012). Update on PML and PML-IRIS occurring in multiple sclerosis patients treated with natalizumab. J Neuropathol Exp Neurol.

[33] Bharucha-Goebel DX, Norato G, Saade D (2021). Giant axonal neuropathy: cross-sectional analysis of a large natural history cohort. Brain.

[34] Dong-Si T, Richman S, Wattjes MP (2014). Outcome and survival of asymptomatic PML in natalizumab-treated MS patients. Ann Clin Transl Neurol.

[35] Dong-Si T, Gheuens S, Gangadharan A (2015). Predictors of survival and functional outcomes in natalizumab-associated progressive multifocal leukoencephalopathy. J Neurovirol.

